# An efficient and secure protocol for checking remote data integrity in multi-cloud environment

**DOI:** 10.1038/s41598-021-93073-3

**Published:** 2021-07-02

**Authors:** H. Anwarbasha, S. Sasi Kumar, D. Dhanasekaran

**Affiliations:** 1 Department of Computer Science and Engineering, Saveetha School of Engineering, Saveetha Institute of Medical and Technical Sciences, Chennai, India; 2 Department of Computer Science and Engineering, Saveetha Engineering College, Chennai, India

**Keywords:** Computational science, Computer science, Information technology

## Abstract

In cloud environment, huge quantity of data has been generated at each and every second. In order to manage the data, cloud service provider makes use of multi-cloud platform to fulfill the requirement. The service provider cooperatively operates altogether for the accessibility of resources and are improvised by implementing the dynamic operation that can run at a time through the Multi-cloud environment. This paper presents a Dynamic Level Based Integrity Checking Protocol (DA-ICP) for storing data in multicloud environment. The proposed method introduces Provable Data Possession (PDP) approach which enables a user who outsources the data at an untrusted multi-cloud for ensuring that the server possesses the original data without downloading it. This model creates a probabilistic proof of possession by sampling an arbitrary collection of blocks from server that considerably minimizes the cost. The effective and secured outsourced data has been resolved using public key cryptography and undergo encryption using Efficient-PDP (EPDP). During experimentation, the presented DA-ICP shows a maximum accuracy of 96.78%. The proposed method uses Multi-cloud in DA-ICP which produces an efficient output than other existing techniques.

## Introduction

**Figure 1:**
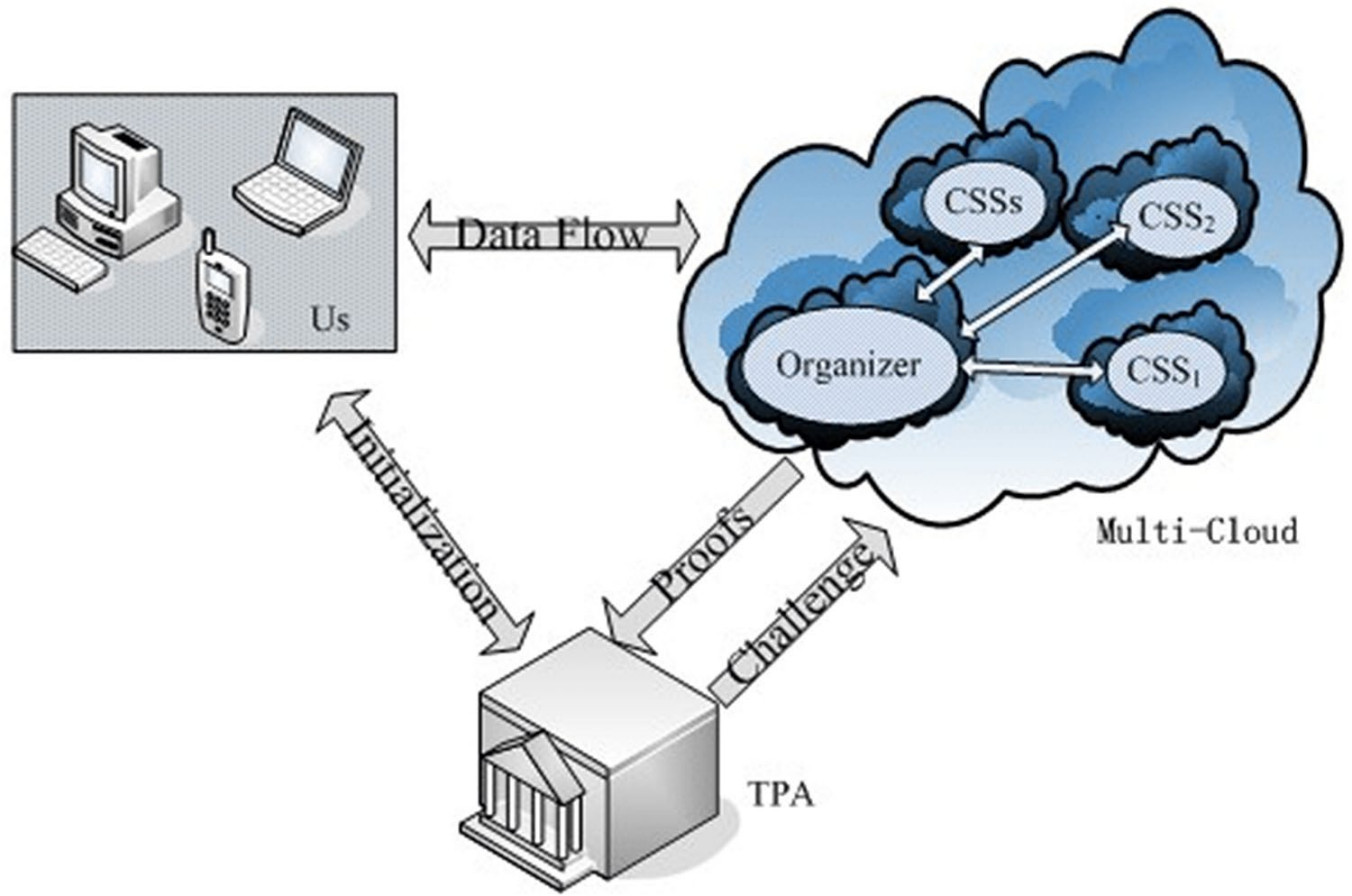
Multi-cloud architecture.

In cloud computing, the cloud storage service is growing increasingly nowadays as significant cloud service type, whereas the stake holders might store the data remotely into the cloud^1^. The stake holder can make use of service on-demand high quality storage through shifting the data to cloud which offers through the cloud service providers. It is highly advantageous to accumulate the data in cloud. But, if the data is deployed in an unreliable cloud, it might simply get corrupted or lost because of the human errors and hardware failures. Sometimes, the corruption of server or disruption might occur with main providers of the cloud service. It might eliminate the rarely accessed or unused data to conserve the space and it claims that all the data is perfectly stored still within the cloud otherwise it hides the loss of data occurrences in order to manage the reputation. In a distributed way, the stake holders save the data with a view to protect the data. The data that is saved in multi-cloud might minimize the data availability and integrity to some extent. The architectural model of multi-cloud storage is displayed in Fig. 1. But, there is no powerful metric to make the stake holder that the data are secure in clouds. The stake holders can validate the integrity of data conventionally by themselves. But, it is unsuitable to let either users or service providers to perform those validations in cloud storage system as none can be assured to give unbiased validating result. The validation by third party is an appropriate option in this case. When comparing with normal users, it comprises a highly powerful communication and computation capabilities. The stake holders might be facilitated and relived the integrity checking of the data saved in cloud through the validation of third party auditor. There exist numerous significant needs for the third party auditing in cloud storage models.

To validate the outsourced data integrity, numerous protocols has been projected at present times. For instance, a protocol named provable data possession (PDP) allows the validating party for ensuring the data integrity publicly. But, this protocols aims over a single cloud, that are unsuitable and inefficient for multicloud circumstances as numerous of it are built for data integrity checking over single user single data file. A third party checker might get numerous checking delegations from various stake holders with the extensive cloud computing adoption. The efficacies would be enhanced greatly and the communicating cost become decreased when the numerous tasks might be managed in a batch way. But, some protocols are projected for batch auditing. The batch auditing will be a suitable one while the entire stake holder community challenged signatures and data blocks are saved perfectly as grouping the numerous proofs over unique data files from unique user into single. The batch auditing will be considered as failure one, when any data signature or data block had been eliminated or destroyed. Therefore, the corrupted data identification may not be attained from batch auditing. A binary search technique may be employed to additionally recognize the corrupted data that is not an efficient manner as redundant steps of auditing are required.

To build the approach for data integrity checking, many studies have been performed in a multi-cloud and conventional cloud environment. Some studies have been reviewed that are relative to the projected technique. With community client repudiation^2^, make sure the public reliability. By employing verifier-local and vector commitment revocation group signature, they demonstrated the collusion attacks in standard techniques and provide an effective approach for public integrity checking with revocation of secure group user. There is a possibility that anyone of client might comprise malignant data in the group. In client’s data that are grouped, security is the major issue while a semi-confined cloud server works together, security is the major issue. The projected process aids at group data encoding and decoding procedures among the repair process of data and gives secure and capable customer denial. The experimental results give that the projected model is secure and better.

For shared data integrity^3^, represents a public auditing method with well-organized user revocation. The cloud computing is enabled to re-signatures the blocks through using the intermediary re-signatures technique for presented users over client repudiation span with the aim that the current client is not requirement to derive re-sign and get back blocks. An auditor looks continuously the collected data pragmatism without need to recover the whole data sum from the cloud. Through ensuring numerous auditing procedures simultaneously, the projected method contains the capability to data auditing in batch manner.

To carry out collusion resistance, multi-user modification, and computation cost, an approach of data integrity validation is presented in^4^ for the clients. The plan depends on the technique of embraces intermediary label update and polynomial affirmation that aids in dynamic client denial and public reviewing. Most Significant Index Generation Technique (MSIGT) is recommended^5^ that improve viable and secured token generation duration using Most Significant Digit (MSD) radix sort. For generation of secured token, an analytical system is enhanced to denote keyword encoding. In the server-supported system, a plan of convertible Identity Based Encryption (IBE) is presented^6^. It releases almost the entire methods relating to key creation towards a Key Update Cloud Service Provider. Through using novel techniques of conspiracy resistant or secret, the target is done. The key distribution process is reasonably longer. In RBAC structures, it aids for flexible resource encryption.

An approach of viable verifiable data ownership is projected through^7^ which employ a chunk vector count and sequence number to aid in modification of data block. With minimized estimation, communication overhead and storage stockpiling this technique is used. The secrecy assurance, multiple-duplicate reviewing, public checking, and authenticity are the confinements that are not assisted. For distributed stockpiling, a security-assurance checking rule is used^8^. To manage the data owners to save the data accurately, a secure and proficient dynamic auditing method is constructed^9^ in cloud. To offer data dynamic functions, the projected model^10^ is expanded subsequently to save the data in cloud efficiently which are secure and efficient in random oracle approach. The experimental results demonstrated that the auditor computation time is minimized.

For ensuring the data integrity, for CSP, a technique of PDP is projected without whole data downloading. The plan security is represented and found that the technique of multi-prover zero-knowledge validation is found to be advantageous for the purpose of knowledge soundness, zero-knowledge and culmination. The approach of non-private or public integrity auditing has been projected^11^ to support the dynamic data distribution with numerous user managing environments. A security ensured arrangement is projected through^12^ which help in the action of dynamic data. With no support from TPA, it provides public verifiability. The projected method manages the security further on TPA. The simulation outcome demonstrated that the projected technique is effectual.

In real-time probable applications, ensuring data auditing with no outflow of data is the main issue. For data privacy^13^, presented the zero-knowledge proof technique for data privacy to denote the data integrity audition that make sure that the validating individual cannot derive any additional data. IND-Privacy and identical-game-related elucidation are suggested^14^ to compute the privacy of data preserving over cloud. They came to a finding that the method of IND-Privacy definition innumerable is not secure hypothetically. They had given a test protocol depending on IND-Privacy which ensures the validation of content-integrity. The validating individual and coordinator might reduce the computational adaptability and correspondence overhead for auditor. In^15^, the protocol is said to be defenceless. Any coordinator or malevolent CSP might create reaction which might pass through validation when eliminated entire data. It does not comprise the soundness guarantee. Identity-based distributed provable data possession-ID DPDP plan and are demonstrated through^16^. This is secured provably one in standard CDH problem supposition in addition to the common verification and assigns public checking and validation.

In multi-cloud data storage, for validating data integrity, a new remote technique is suggested^17^ named as identity based distributed PDP. From the standard Computational Diffie-Hellman (CDH) problem, the projected ID-DPDP protocol is effective and flexible with certificate management elimination, the projected ID-DPDP protocol restrained. This protocol realizes public verification, delegated verification and private verification from client’s standpoint. In purview of hierarchy and homomorphic evident response, an agreeable PDP technique is presented. With multi-proved zero-knowledge proof systems, this study proofs the security plan which can fulfil culmination, zero-knowledge aspects and knowledge soundness.

Few of the protocols are based on RSA type of public key cryptography. Some related developments in quantum computing that may present threat to these cloud service, and on the other hand, it provides opportunities in these services. For instance, RSA public key protocol will be insecure if quantum computer is available^18^. There are now new ways for generating random numbers such as quantum random generation^19,20^, unclonable physics device^21^. The analysis depicts the solution demonstrates low correspondence and computation overheads when comparison of non-agreeable technique. There exist a necessary to construct efficient techniques for data integrity checking for multi-cloud environment.

In this paper, a Dynamic Level Based Integrity Checking Protocol (DA-ICP) is presented to place the data in the multi-cloud platform. The DA-ICP develops a model for PDP which enables the client whom outsourced a data at an untrusted multi-cloud to ensure that the serve holds the actual data with no need of downloading. This model creates a probabilistic proof of possession by sampling an arbitrary collection of blocks from server that considerably minimizes the cost. The effective and secured outsourced data has been resolved using public key cryptography and undergo encryption using Efficient-PDP (EPDP). It enables the dynamic data which effectively offers various operations such as block modification, deletion and append. To further improve the efficiency of the presented model, Linear congruential generators is used for random sequence generation.

The upcoming portions of the study are planned here. “Proposed data integrity checking protocol” and “Security analysis” briefs the presented model. “Data dynamics” provides the security analysis and the experimental details are given in “Performance validation”. “Conclusion” offers the conclusion.

## Proposed data integrity checking protocol

**Figure 2:**
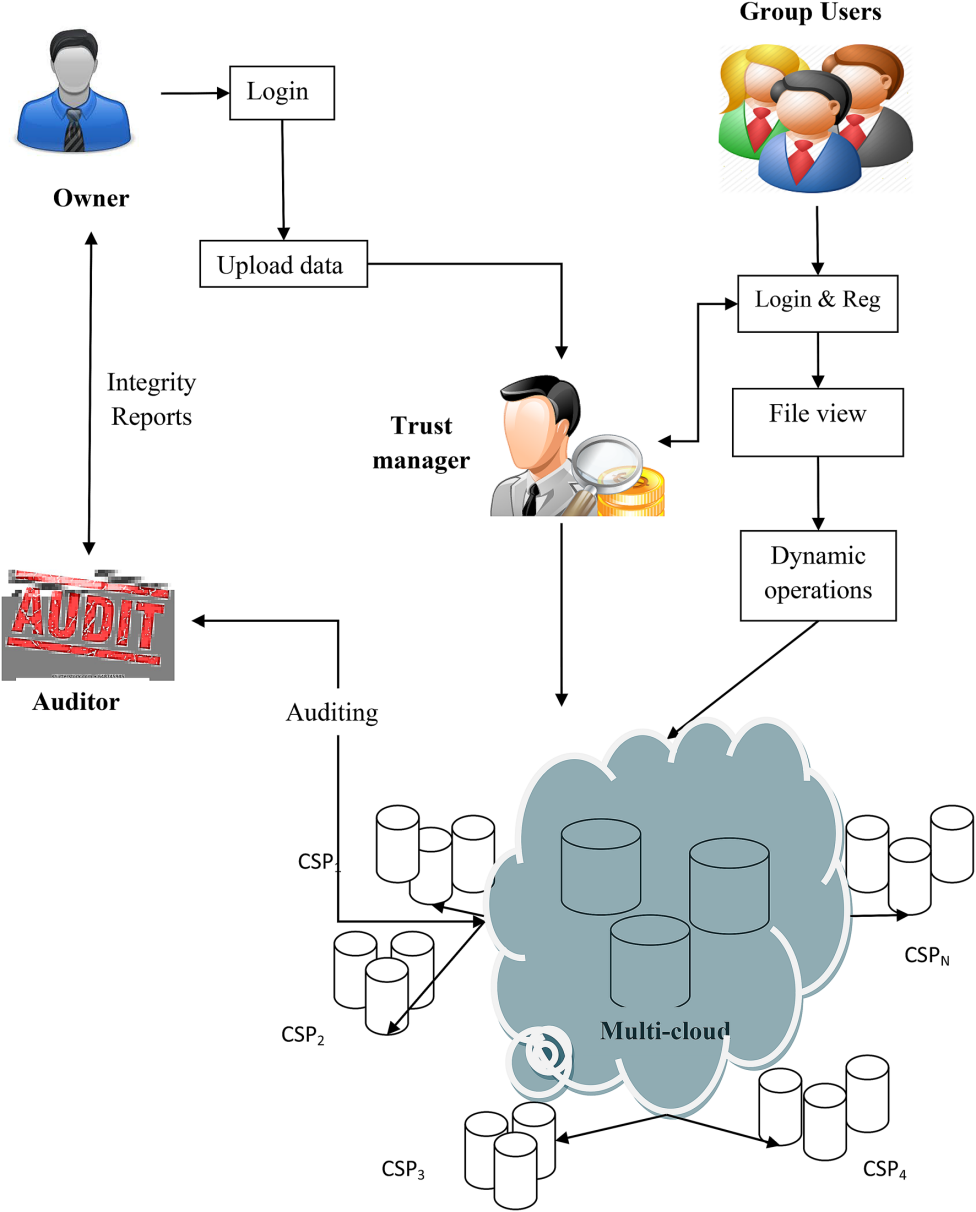
Architecture of data integrity checking protocol.

A cloud storage system is assumed that has an un-trusted server and a user. The user stores the data in server with no local duplication. Therefore, the user has to validate the data integrity which is store in un-trusted server that is remote and it is essentially significant one. The user has to identify when the server changes any user’s data partition. Additionally, any individual must be capable to recognize it. The data should be stored private if a third party verifier validates for the data integrity. An architecture of the data integrity checking technique is shown in Fig. 2. We provide an agreement among the server and client in this method. The detection data is selected by probability which minimizes the computation at both verifier and server end. A set of five stages are comprised in the projected method: Setup, Linear congruential generation, Siggen, Agreement, Challenge, and Verification.

$H~\left( \cdot \right),h\left( \cdot \right)$ refers the cryptographic hash function.

In common, we employ standardized hash functions.

$f~\left( \cdot \right)$—Linear congruential generator function.

$P_{X}$—the probability for making sure the modified data blocks detection.

$t$—consider that the data blocks count that have been modified.

$N_{a}$—refers the random number.

### Setup

Primarily, $k$ is the security parameter. The method of key generation is executed by the user and it returns a secret key $sk$ and public key $pk$ that are matching. When the secret key $sk$ is maintained secretly through the user, public key $pk$ is open to everyone. The user chooses authentication index $r$ and $f~\left( \cdot \right)$ as Linear congruential generator function.

In the un-trusted server, $m$ demonstrates the file that is saved is segmented into $n$ equivalent lengths blocks $m~ = ~\left\{ {~m_{1} ,m_{2} ~, \ldots ,~m_{n} } \right\}$.

### Linear congruential generation

There exist numerous random number generator (RNG) families: multiple recursive, linear congruential, “computer operation” techniques etc., Transfer function are comprised in a linear congruential generator of the below kind.

1 $$f\left( x \right) = \left( {ax + c} \right)mod~m^{1}$$

where $a$ refers the multiplier, $c$ refers the increment and modulus is the $m$ and $x,~a,~c,~m~ \in ~N$. $f$ is $x_{n} = \left( {ax_{{n - 1}} + c} \right)mod~m$.

Specially, $c$ and $m$ are selected to be prime relatively and a $\forall x~ \in ~N$, $ax~mod~m~6 = ~0$. The linear congruential generators cycle length will go beyond modulus $m$, but can maximized with three below constraints.

- increment $c$ is prime relatively to $m$
- $a - 1~$ refers a multiple of each prime segmenting $m$,
- $a~ - ~1$ refers a multiple of 4 whenever $m$ is a multiple of 4.

While $c~ = ~0$, Lehmer algorithm or ParkMiller algorithm special case. Let with $a^{j} ~mod~m$ denote, $n~ + ~j$ th term might be simply expressed from $n$ th term. At last, we employ commonly three output function kinds:

2 $$g:{\mathbb{N}} \to \left[ {0,1} \right[,~and~g\left( x \right) = \frac{x}{m}$$

3 $$g:{\mathbb{N}} \to \left] {0,1} \right],~and~g\left( x \right) = \frac{x}{{m - 1}}$$

4 $$g:{\mathbb{N}} \to \left] {0,1} \right[,~and~g\left( x \right) = \frac{{x + 1/2}}{m}$$

In the $R$ function $congruRand$, Linear congruential generators are executed.

### SigGen

For every block, the client estimates the validation tag. For tag encryption, ${{\sigma }}_{{\text{i}}} = \left( {V_{i} } \right)_{{SK}} ,\phi = \left\{ {{{\sigma }}_{{\text{i}}} } \right\}, \,1 \le i \le n$ denotes the entire tag set and transmits $\left\{ {m,\left( {i,\sigma _{i} } \right)} \right\}$ towards the server, $V_{i} = H\left( {h\left( {m_{i} } \right),r} \right),~~~1 \le i \le n,$ employing $sk$ as secret key.

### Agreement

The data blocks probability information is sent through the user to detect the $\left\{ {V,n,P_{X} ,t,M_{a} } \right\}_{{C^{{ - 1}} }}$ verifier. The data is received through the verifier and employs client public key for decrypting the data. After decryption to estimation to be recognized for c data block, towards the client $\left\{ {C,n,P_{X} ,t,N_{a} + 1,C} \right\}_{{V^{{ - 1}} }}$ is sent. The message is decrypted by the received client to make sure that the verifier gains a message from the user.

### Challenge

The c data blocks are chosen by the verifier that are subjected to detect and on the other hand, for every data block, the arbitrary challenge $C_{i} = f\left( i \right),1 \le i \le n$. $Chal = \left\{ {c,C_{i} } \right\}$ is sent to the verifier towards the server.

### Verification

The server estimates $h\left( {m_{j} } \right),1 \le j \le c$ when the message is received which is from verifier.

5 $$K = C_{1} h\left( {m_{1} } \right) + C_{2} h\left( {m_{2} } \right) + \cdots + C_{C} h\left( {m_{c} } \right)$$

The server derives $\sigma _{i}$ and returns $\left\{ {K,\sigma _{i} ~\left( {~1 \le ~i \le ~c} \right)} \right\}$ to the verifier to decrypt $\sigma _{i}$ whereas $V_{i} = \left( {\sigma _{i} } \right)_{{PK}}$ to estimate.

6 $$R = ~C_{1} V_{1} \oplus C_{2} V_{2} ~ \oplus \ldots \oplus ~C_{2} V_{2} {\text{and }}R^{\prime} = ~H\left( {K,~r} \right)$$

**Figure 3:**
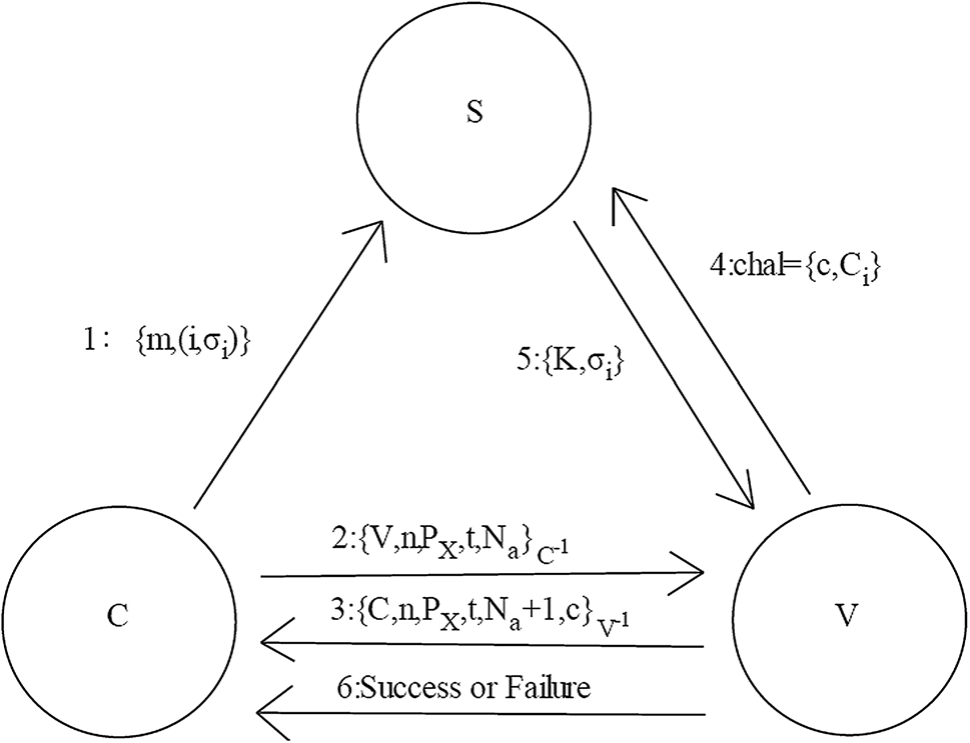
Flowchart of proposed model.

The verifier validates for the condition $R = R'$.The function display as “success” when the condition is true or “failure”. The flowchart protocol is demonstrated in Fig. 3.

## Security analysis

Security is defined as the protocol is secure over the un-trusted server and it is private against at third-party verifiers. The user cannot pass validation until it should access entire unchanged $m$ version. Primarily, it is considered that the remote server is in un-trusted state; it can unintentionally or intentionally modify the user's data.

### Lemma 1

For a secured hash function, the probability is insignificant, the attacker $X$ can search with the file 'm' that comprising a similar function rate and is varying from m:

7 $$prob\left[ {\left( y \right) \leftarrow X\left( {x,h\left( x \right)} \right):y \ne x,h\left( y \right) = h\left( x \right)} \right] < \varepsilon$$

### Theorem 1

A secured hash functions H(), h() and secured private key encryption is employed by the projected protocol in order to lemma 1, the attacker X might interfere with success probability and data that is insignificant are also known.

### Proof

In query outcomes, consider that the un-trusted server $m_{i}$ interfered and also estimates $h(m^{\prime}_{i} ),~\because ~m^{\prime}_{i} \ne m_{i}$ in order to lemma.

8 $$prob[h\left( {m^{\prime}_{i} } \right) = h\left( {m_{i} } \right) < \varepsilon$$

Through the hash function nature, it is similar as $prob\left[ {R^{\prime} = R} \right] < \varepsilon$. Thus, the attacker can interfere with success probability that is insignificant and data. During verification stage, it is significant to denote that the $\sigma _{i}$ is decrypted through verifier and there is insignificant probability in deriving message $m_{i}$, therefore, this aids in validation of third-party security. The message is transmitted by the adversary’s forgery signature probability that is small and private key signature for the agreement phase security. Additionally, secret data does not get included in the message. To perform data integrity verification, agreement phase is making sure by the authorized person.

## Data dynamics

By the process of data insertion (I), data append (A), data modification (M) and data deletion (D), the method efficiently handles the complete operation of dynamic data. We consider that the signature φ and file m is store and generated at server in dynamic operation design.

### Data modification

In cloud data storage, data modification is the primary stage that is the highly used operation. The fundamental operation of data modification refers to certain blocks replacement with fresh ones. Primarily, the client produces the respective signature $\sigma ^{\prime}_{i} = \left( {H\left( {h\left( {m^{\prime}_{i} } \right),r} \right)} \right)$, if the user need to change the i-th block $m_{i}$ towards $m^{\prime}_{i}$. In order to the update procedure “$update = \left( {M,i,m^{\prime}_{i} ,\sigma ^{\prime}_{i} } \right)$” is the update request message and transmits to server, wherever M demonstrates the modification operation.

The server execute $ExecUpdate\left( {m,\phi ~,~update} \right)$ when request is received. The server replaces the block $m_{i}$ with $\sigma _{i}$, $\sigma _{i}^{'}$, $m_{i}^{'}$ especially and estimate $h\left( {m_{i}^{'} } \right)$. For $\left( {i,h\left( {m_{i}^{'} } \right),\sigma _{i}^{'} } \right)$ operation, the server response towards the user. The user primarily decrypts $\sigma _{i}^{'}$ after deriving modification operation proof from server and validate that $\left( {(\left( {\left( {H\left( {h\left( {m^{\prime}_{i} } \right),r} \right)} \right)} \right)_{{Sk}} } \right)_{{pk}} = \left( {H\left( {h\left( {m^{\prime}_{i} } \right),r} \right)} \right)$, when it is satisfied displays failure or displays true.

### Data insertion

In this operation, data is added to the accessible block and are precisely similar with the data modifications. This paper assumes the data files might be allocated to one or additional block data that exist for the single data block insertion and carry out operation of data modification.

### Data deletion

It is the data insertion conflicting operation. For eliminating block $m_{i}$, if the server gets the update request, it might be replaced with DBlock the block $m_{i}$ that it is a fixed special block which demonstrates the deleted blocks. The protocol process is same as the data insertion and modification.

### Data append

Appending the data does not modify the steps when comparing with data modification. A new data blocks $m_{j}$ is inserted at some position where $j = n + k,k \in N^{*}$, as the respective message of update request is “$~update~ = ~\left( {A,~j,~m_{j} ,\sigma _{j} ~} \right)$”, wherever modification operation is denoted through $A$.

## Performance validation

In Multi-cloud computing environment, the integrity of data is the highly hot security issue. In this study, the importance of data integrity is taken into consideration. And, an examination of unique conventional data integrity techniques is done where the advantages and drawbacks are determined. A comparison among the conventional and proposed methods is made. To control the data in remote cloud, different clients are allowed by the projected method. In order to verify, inquiry and update time cost, the projected method implementation is validated. While comparing with other technique, the update time cost rate is lower. Because of the signature integrity verification approach, time cost value is high. Through comprising a strict verification rule and sensitive update time, this make sure the fixed security and quality of service. Table 1 provides the comparative results of the existing and proposed methods interms of accuracy and time.

**Table 1:** Comparison of proposed and existing methods interms of accuracy and time.

S. No	Techniques	Accuracy (%)	Time (ms)
1	Proposed	96.78	2.01
2	Rank-based authentication skip list	89.00	2.50

**Figure 4:**
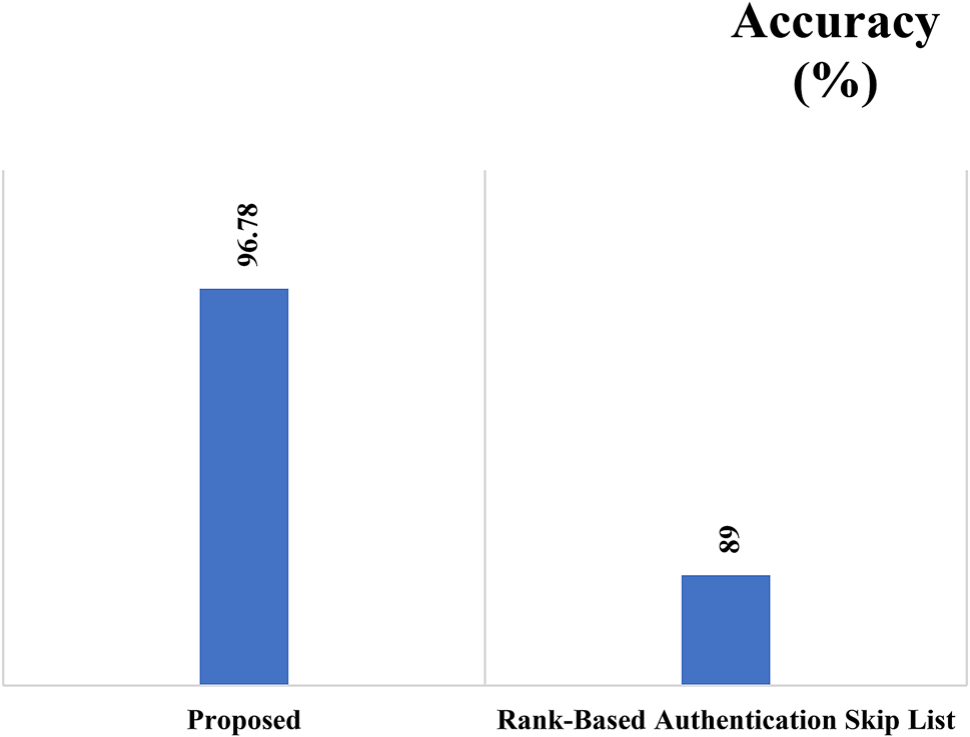
Accuracy analysis.

**Figure 5:**
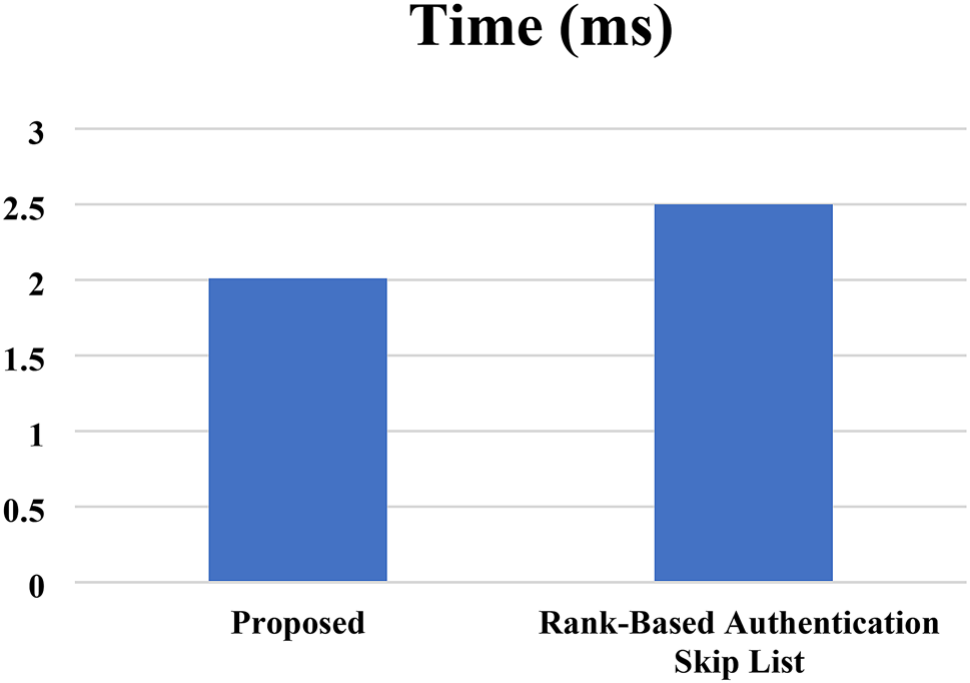
Execution time analysis.

Figures 4 and 5 depicts the simulation results of the integrity checking protocol implemented to examine the projected method with the recently existing method known as rank-based authentication skip list interms of accuracy and time. While comparing to the existing method of rank-based authentication skip list, the presented method gives superior results. By means of accuracy and execution time, the projected technique is compared and is shown in the table. The execution time is measured by means of milliseconds (ms) and the accuracy is measured by means of percentage. The rank-based authentication skip list method attains 89% of accuracy. The projected method attains 96.78% as accuracy and it shows that the projected method superior by means of accuracy. For execution time, rank-based authentication skip list method takes 2.50 ms whereas the projected method takes only 2.01 ms. It is absolute from the figure that the projected method attains enhanced rate by means of execution time and accuracy.

## Conclusion

This paper has presented a DA-ICP to store the data in multi-cloud environment. It enables the dynamic data which effectively offers various operations like block modification, deletion and append. To further improve the efficiency of the presented model, Linear congruential generators is used for random sequence generation. The rank-based authentication skip list method attains 89% of accuracy. The projected method attains 96.78% as accuracy and it shows that the projected method superior by means of accuracy. For execution time, rank-based authentication skip list method takes 2.50 ms whereas the projected method takes only 2.01 ms. The simulation results reported that the presented method offers efficient results over the compared methods.
